# Identification of population of bacteria from culture negative surgical site infection patients using molecular tool

**DOI:** 10.1186/s12893-020-01016-y

**Published:** 2021-01-07

**Authors:** Himanshu Sekhar Behera, Nirupama Chayani, Madhusmita Bal, Hemant Kumar Khuntia, Sanghamitra Pati, Sashibhusan Das, Manoranjan Ranjit

**Affiliations:** 1 ICMR-Regional Medical Research Centre, Bhubaneswar, 751023 Odisha India; 2 Department of Molecular Epidemiology, ICMR-Regional Medical Research Centre, Bhubaneswar, 751023 India; 3 Department of Microbiology, SCB Medical College and Hospital, Cuttack, 753003 India; 4 Department of Parasite Immunology, ICMR-Regional Medical Research Centre, Bhubaneswar, 751023 India; 5 Department of Public Health, ICMR-Regional Medical Research Centre, Bhubaneswar, India

**Keywords:** Surgical site infections, Culture negative surgical site infections, PCR assay in SSI, Broad-range 16S rRNA gene PCR for SSI, Unculturable bacteria, Anaerobic bacteria

## Abstract

**Background:**

Managing surgical site infections, with negative culture report in routine diagnosis is a common dilemma in microbiology accounting more than 30% worldwide. The present study attempted to identify the presence of bacterial *spp.* if any in wound aspirates/swabs of culture negative surgical site infections of hospitalised patients using molecular tools.

**Methods:**

Ninety-seven patients with post-operative SSI whose wound swabs/aspirate were negative in the conventional aerobic culture after 72 h of incubation were analysed by 16S rRNA gene specific broad range PCR. The amplified DNA fragments were sequenced by Sanger DNA sequencing method and homology of the sequence were matched using NCBI BLAST (NCBI, USA)

**Results:**

Of the 97 patients, 16S rRNA based broad range PCR assay could identify the presence of bacterial pathogen in 53(54.63%) cases, of which 29 isolates were supposed to be of viable but non-culturable bacteria (VBNC), 07 were of obligatory anaerobes and 13 were of unculturable bacteria, 04 were with poly bacterial infections.

**Conclusions:**

Our study highlights the usefulness of PCR assay in detecting the presence of any VBNC, anaerobes and unculturable bacteria in SSI patients regardless of how well the bacteria may or may not grow in culture. Measures should be taken to use anaerobic culture system and PCR diagnosis along with conventional culture to detect the VBNC and unculturable bacteria where Gram stain is positive for better patient care.

## Background

Maintaining and improving the quality in healthcare service is a major concern in hospitals and other health care facility. Surgical site infections (SSI) are the most common post-operative infections of skin or underlying soft tissue that sometimes causes postoperative morbidity, mortality, increase hospital costs and prolongs hospital stay [1]. SSI is the third most commonly reported nosocomial infections after ICU infections and urinary tract infections (UTI) in a hospital set up approaching 14–16% of all nosocomial infections and 2.5% to as high as 41.9% of nosocomial infections in surgical patients [2, 3]. In developing countries, around 5.6% of all surgical procedures develops SSI, but in India incidence of SSIs consistently shown higher rates ranging from 23 to 38% [4, 5]. As per Centre for Disease Control and Prevention (CDC) and the European Centre for Disease Prevention and Control (ECDC), SSI is defined as, “postoperative infection occurring within 30 days of surgery or within one year if any prosthetic material is implanted at the surgical site” [6]. There are some ways by which surgical sites can get infected such as: use of unsterile instruments, contaminated prosthetics/surgical solutions while performing surgical procedures, improper cleaning of surgical site by infected surgical solutions. These activities might allow entering of skin flora such as *Staphylococcus epidermidis, Staphylococcus aureus, Mycoplasma* species and microbial flora of gastrointestinal tract and the colon in particular that include *Escherichia coli, Clostridium perfringens, Bifidobacterium, Enterococci, Lactobacilli, Bacteroides and Helicobacter pylori *etc*.* into the surgical site, which later causes infections [7, 8].

Culture negative surgical site infection is a common problem while furnishing a report in a microbiology laboratory, which is defined as “a patient with all the clinical signs of surgical site infection, but with “no bacterial growth” in the conventional culture [9]. Incidence of such ‘culture negative SSIs’, as reported in some of the studies can be up to 30%, where cultures do not exhibit bacterial growth even when clinical signs of infection are present [10]. Most anaerobic bacterial colonies often do not grow in routine culture media. Fastidious bacteria with special nutritional requirements (e.g., *Propionibacterium acnes*) mostly remain undetected in routine culture media unless their special nutritional requirement being compensated [11]. Apart from these factors, sample collection after antibiotic treatment for prolong period, presence of viable but non-culturable (VBNC) bacteria or presence of unculturable bacteria in sample also leads to culture negative SSIs. Sometimes the culture plate remains sterile after 72 h of incubation due to the presence of biofilm production by bacteria.

The viable but non-culturable (VBNC) state, a special physiological state of bacteria was first discovered and presented by Xu et al*.* in 1982, after that several studies reported the presence of these VBNC bacteria in a variety of clinical samples [12, 13]. The VBNC state is slightly different from dormancy state where they exhibit measurable metabolic activity, but don’t grow in conventional culture medium like normal bacterium [14]. Studies have reported 85 species of bacteria can enter the VBNC state, including 18 non-pathogenic species and 67 pathogenic species [14]. As only aerobic culture system is available in most of the microbiology laboratories, the anaerobic bacteria present in sample cannot grow in that aerobic conditions and their presence remain hidden even after 72 h of incubation [15]. Sometimes the culture media or growth conditions are not supportive for the growth of some bacteria broadly named as unculturable bacteria, hence no bacterial growth is noticed even after the incubation of plate for 72 h [16]. Those unculturable bacteria grow in their natural environment, but don’t grow in standard laboratory conditions, still they cause infections. Several studies had also reported the presence of unculturable bacteria in clinical specimens [16, 17].

Conventional culture is the Gold standard to identify the causative bacteria in clinical samples, but the results are completely dependent on the presence of viable organism and time of processing of samples after collection. There is always a need for a broad spectrum, rapid diagnostic method to detect and identify the causative bacteria from a symptomatic person, if the Gram stain is positive but culture is negative even after 48/ 72 h of incubation. After the invention of PCR, molecular diagnosis was attempted with PCR for a rapid and accurate diagnosis of all diseases. DNA-based molecular approach is used to identify exact bacterial aetiology if any biofilm producing bacteria or VBNC bacteria or any unculturable bacteria is present in clinical samples of a symptomatic person with Gram stain positive but no growth in culture. Sometimes, it is observed that, multiple bacteria or bacteria with fungi may exist within the clinical samples but cultures only detect a small fraction of these pathogens, but PCR with pyrosequencing can identify those causative pathogens. These findings lead to dramatic improvements in treatment. Broad-range 16S rRNA gene specific PCR assay is useful in identifying the causative pathogen even in polybacterial infections, presence of fastidious, unculturable or VBNC bacteria in sample [18].The present study was carried out to know the presence and frequencies of various bacteria in wound aspirates/ wound swabs of few Gram stain positive but culture negative surgical site infection patients attending orthopaedics and gastroenterology department of a tertiary care hospital of Odisha, an eastern India State using molecular tools.

## Methods

### Study design

This was a prospective, nonrandomized study carried out at ICMR—Regional Medical Research Centre, Bhubaneswar in collaboration with SCB Medical College, Cuttack, Odisha from September 2017 to June 2019. The aim of the study was to find the presence and frequencies of various bacteria in pus/ wound swabs of some culture negative surgical site infection patients attending a tertiary care hospital (SCB Medical College, Cuttack) of Odisha, an eastern India State using molecular tools. The study was funded from Science and Engineering Research Board (SERB) of India.

### Ethics approval and consent to participate

This study was approved by the Research and Ethics Committee of Regional Medical Research Centre (RMRC), Bhubaneswar, Odisha (Ref No. ECR/911/Inst/OR/2017). The informed consent was obtained from patient. Research methodology followed was adhered to the tenets of the Declaration of Helsinki.

### Patient selection

Patients with postoperative SSIs that developed within 30 days of surgery with signs of inflammation, such as edema, redness, warmth, fever exceeding 38 °C, and pus/swab samples that were negative in conventional aerobic culture but positive in Gram stain were included in the study. As only conventional aerobic culture facility is available in SCB Medical College, Cuttack, Odisha, the samples (pus/ swab) which comes negative after the culture were brought to ICMR- Regional Medical Research Centre, Bhubaneswar for further molecular identification. Surgical site infection patient’s samples (swabs/ aspirates) found to be positive in conventional aerobic culture after 72 h of incubation at SCB Medical College, Cuttack, Odisha were excluded from this study.

### Collection of clinical specimens

A total of 97 culture-negative samples (pus aspirates/swabs) were collected from the Microbiology department, SCB medical college, Cuttack aged 28 to 84 years with postoperative SSIs were transported to the RMRC laboratory for further processing and analysis using molecular tools. All the samples were collected from orthopaedics and gastroenterology department after any surgical procedures. All patients had been treated with antibiotics before the specimens were taken for Gram staining and culture.

## Molecular diagnosis

### DNA isolation and Broad-range PCR assay

DNA was extracted from the pus/ wound aspirate specimens using commercial QIAamp DNA Mini Kit (Qiagen), as per the manufacturer’s instructions. Briefly, broad-range PCR assay was standardized to amplify ~ 1492 bp region of 16S rRNA gene using published primers (FP: (27F) 5′-AGAGTTTGATCCTGGCTCAG-3′ and RP: (1492R) 5′-GGTTACCTTGTTACGACTT-3′) while varying the annealing temperature and conc. of MgCl2 [19]. PCR amplification was carried out in 25 μl of final reaction volume containing 1 × reaction buffer (Fermentas), 0.2 mM dNTPs (Fermentas), 0.40 μM of each primer (IDT) and 1.25U Taq polymerase (Fermentas). The temperature profile of the PCR assay was as follows: initial denaturation for 04 min at 94 °C, followed by 35 cycles of denaturation for 1 min at 94 °C, primer annealing for 1 min at 57 °C, strand elongation for 1 min at 72 °C, with the final elongation for 10mins at 72 °C temperature. DNA isolated from known isolates of *E. coli* was used as a positive control and reaction mixture with 5 μl of distilled water was used as a negative control in all PCR reactions. Amplified PCR products were electrophoresed on 1.0% agarose gel and visualized under a gel documentation system (Syngene).

### Nucleotide sequencing and homology analysis

Amplified DNA bands from 16S rRNA gene based PCR assay were cut with sterile scalpel bladesfrom the agarose gel and processed for Sanger nucleotide sequencing (Eurofins). The obtained nucleotide sequences were searched for homology analysis with the available sequences of 16S rRNA gene of the GenBank using NCBI BLAST (NCBI, USA) computer program (http://www.ncbi.nlm.nih.gov/pubmed). Nucleotide sequences of samples which showed > 90% homology with the available 16S rRNA sequences of any bacteria were submitted to NCBI to obtain the respective accession numbers.

## Results

Of the ninety-seven (97) culture negative surgical site Infection (SSI) patient samples, 16S rRNA based broad range PCR assay could able identify the presence of bacterial pathogen in 53(54.63%) cases, all of which were successfully sequenced through Sanger sequencing. Of the 53 nucleotide sequences, (n = 12;22.2%) were found belongs to *Bacillus* spp., (n = 13; 24.07%) were uncultured bacterium, (n = 06; 11.1%) *Pseudomonas* spp., (n = 06; 11.1%) were of *Enterococcus* Spp., (n = 02; 3.7%) were *Bacteroides*, (n = 02;03.7%) were *Fussobactrium* sp., (n = 02; 3.7%) *Massilia* sp., (n = 01; 01.8%) *Staphylococcus* sp., (n = 01; 1.8%) *Sneathia* sp., (n = 1; 1.8%) *Peptostreptococcus* spp., (n = 1; 1.8%) *Kleibsiella* sp., (n = 1; 1.8%)*Stenotrophomonas* sp., (n = 1; 1.8%) *Peptoniphilus* sp., (n = 4; 7.4%) samples were of multiple bacterial infections (as per Sanger sequencing results). Of the 53 samples positive for PCR assay, 49 were submitted to NCBI data bank and obtained respective accession numbers, which are given in Table 1.

**Table 1:** Details and accession numbers of the bacteria identified and submitted to NCBI from culture negative SSI patients

Serial no.	Sample name	Bacteria identified	Accession no.
1	SSI 1	*Bacillus spp.*	MK424355
2	SSI 2	*Bacillus spp.*	MK424356
3	SSI 4	*Staphylococcus spp.*	MK424357
4	SSI 5	*Sneathia spp.*	MK424358
5	SSI 6	Uncultured bacterium	MK424359
6	SSI 7	*Bacillus spp.*	MK424360
7	SSI 9	Mixed infections	NA
8	SSI 10	Mixed infections	NA
9	SSI 11	*Pseudomonas spp.*	MK424361
10	SSI 12	*Bacillus spp.*	MK424362
11	SSI 13	Mixed infections	NA
12	SSI 14	Uncultured bacterium spp.	MK928502
13	SSI 15	*Bacteroides*	MK928506
14	SSI 16	*Fussobacterium spp.*	MK928505
15	SSI 18	*Fussobacterium spp.*	MK928504
16	SSI 20	Uncultured bacterium spp.	MK424363
17	SSI 21	*Bacteroides*	MK928503
18	SSI 22	No significant similarity	NA
19	SSI 24	*Bacillus spp.*	MK424364/
20	SSI 26	Mixed infections	NA
21	SSI 27	*Enterococcus spp.*	MK424365
22	SSI 28	*Pseudomonas spp.*	MK424366
23	SSI 29	*Bacillus spp.*	MK829054
24	SSI 31	*Enterococcus spp.*	MK829055
25	SSI 32	*Massilia spp.*	MK838102
26	SSI 34	Uncultured bacterium spp.	MK934354
27	SSI 35	Uncultured bacterium spp.	MK424367
28	SSI 38	Uncultured bacterium spp.	MK858273
29	SSI 39	Uncultured bacterium spp.	MK424368
30	SSI 40	Uncultured bacterium spp.	MK829056
31	SSI 41	*Peptostreptococcus spp.*	MK858274
32	SSI 42	Uncultured bacterium sp.	MK934355
33	SSI 44	*Bacillus spp.*	MK424369
34	SSI 45	*Bacillus spp.*	MK858266
35	SSI 46	*Enterococcus spp.*	MK829057
36	SSI 48	*Enterobacter spp.*	MK424370
37	SSI 49	*Enterobacter spp.*	MK424371
38	SSI 50	*Massilia spp.*	MK858267
39	SSI 51	*Klebsiella spp.*	MK424372
40	SSI 57	*Pseudomonas spp.*	MK838103
41	SSI 60	*Peptoniphillus spp.*	MK928501
42	SSI 61	*Stenotrophomas spp.*	MK838104
43	SSI 64	Uncultured bacterium sp.	MK934356
44	SSI 66	*Bacillus spp.*	MK829058
45	SSI 68	*Pseudomonas spp.*	MK829059
46	SSI 71	*Pseudomonas spp.*	MK829060
47	SSI 73	Uncultured bacterium spp.	MK934357
48	SSI 76	Uncultured bacterium spp.	MK838105
49	SSI 80	*Pseudomonas spp.*	MK858268
50	SSI83	*Bacillus spp.*	MK858269
51	SSI 87	*Bacillus spp.*	MK858270
52	SSI 91	Uncultured bacterium spp.	MK928500
53	SSI 93	*Bacillus spp.*	MK858271
54	SSI 97	*Enterococcus spp.*	MK858272

Of the 49 identified bacteria, 29 isolates were supposed to be of viable but non-culturable (VBNC) bacteria belonging to *Bacillus* spp., *Pseudomonas* spp., *Enterococcus* spp., *Massilia* spp., *Staphylococcus* spp., *Kleibsiella* spp., *Stenotrophomonas* spp. and 07 were of obligatory anaerobic strains belonging to *Bacteroides* spp., *Fussobactrium* spp., *Sneathia* spp., *Peptostreptococcus* spp., *Peptoniphilus* spp. and 13were of unculturable strains. Out of 49 bacterial isolates identified in this study 21 belongs to Gram positive bacteria and 15 belongs to Gram-negative bacteria. Of all the bacteria 03 were of slow growing(fastidious) nature that are of genus *Massilia* spp. and *Peptostreptococcus* spp. respectively.

## Discussions

Managing SSIs, with negative culture report in routine diagnosis is a common problem in the microbiology laboratory. The frequency of SSI depends upon the type of surgery performed and the hospital environment. Similarly, prevalence of pathogens in SSI varies from place to place and hospital to hospital [20]. Studies reported that, around 5–30% of clinical specimens (wound swabs/ aspirates) isolated from a patient having all clinical signs of SSI, do not show any bacterial growth in conventional culture [10]. In a study conducted in a tertiary care hospital in southern India (Bangalore) 7.8% of all SSIs were culture negative [21]. Similarly, in a study conducted in a medical college in western India (Maharashtra), out of 196 pus samples taken from SSI patients 5.4% were negative in culture [22]. In those cases, although there was the presence of bacteria in some samples, other factors plays a crucial role in preventing their growth on culture plate such as, sample collection after the commencement of antibiotics, presence of viable but non-culturable (VBNC) bacteria, presence of biofilm producing bacteria or fastidious bacteria in the sample [21, 22]. If the laboratory condition is not suitable for the growth of anaerobic bacteria and the unculturable bacteria in samples it can also leads to no growth in culture even though Gram stain was positive.

In this study, we have reported several facultative aerobic culturable bacteria such as *Bacillus spp., Pseudomonas spp., Enterococcus spp., Massilia spp., Staphylococcus spp., Kleibsiella spp., Stenotrophomonas spp.*, from wound swabs/ aspirate of culture negative SSI patients, which points towards their viable but non-culturable (VBNC) state, that results the culture plate turn negative even after an incubation of 48hours. *Bacillus spp.* is a Gram-positive, rod-shaped, facultative anaerobic bacterium commonly found in soil and food of which some strains cause SSIs. In one of the study conducted on postoperative and post-traumatic wounds on 24 patients in an orthopaedic ward of Swedish hospital, mostly *Bacillus spp.*, were reported [23]. *Pseudomonas spp.* is a Gram-negative, rod-shaped, facultatively anaerobic bacterium commonly found in humans, soil and plants. Several studies conducted on SSI patients reported the presence of *Pseudomonas sp.* in different frequencies [24]. Enterococcus *spp.* are the Gram positive, aerobic, cocci that become pathogenic when they colonize niches. In one of the studies conducted on 676 surgery patients, 38(5.6%) were found infected from *Enterococcus spp*.[25]. Similarly, in another study conducted on 2713 SSI patients, *Enterococci* were reported in 46.1% cases, where *E. faecalis and E. faecium* were found in almost equal proportions [26]. *Staphylococcus spp.* is a Gram-positive coccus, facultatively anaerobic, coagulase-negative bacterium that occurs very commonly as a harmless commensal on human skin. Small-colony variants (SCV) are a form of *S. aureus* that grow slowly compared to wild type *S. aureus* and are not recognized by culture even after incubation for 72 h [27]. *Bacteroides* are the Gram-negative, spore forming, obligate anaerobic bacilli. In one of the studies conducted in 2002 role of *Bacteroides* had been reported in SSI patients. *Bacteorides fragilis* has been described as a low-virulence bacterium and has the ability to form biofilm [15]. *Fussobactrium spp.* is a Gram-negative, obligate anaerobic, slender rod-shaped bacillus. *Fussobactrium sp.* and *bacteroides* have the ability to produce certain toxins and enzymes that damage the tissue in SSIs, so influence the pathogenesis of infections [28]. As the laboratory condition was supportive for the growth of only aerobic bacteria, *Bacteroides and Fussobactrium sp.* does not come in the conventional culture even after incubation for 72 h but were identified in PCR. *Sneathia spp* is a Gram-negative, anaerobic, fastidious, rod-shape bacteria. *Sneathia amnii* is recently described as an opportunistic pathogen of oral cavity, intestines and female urinary tract [29], *Massilia spp.* are fastidious, facultatively aerobic, Gram negative rod bacteria. In a study *Massilia spp.* was isolated from a surgical wound infection in a 36-year-old male who had undergone orthopaedic surgery [30]. *Peptostreptococcus spp.* is a fastidious, obligately anaerobic, Gram-positive bacteria. In one of the patients with SSIs, *Peptostreptococcus spp.* was reported in necrotizing fasciitis, infections following trauma and surgical site infections [31] and *Kleibsiella spp*. are Gram- negative, rod shaped, facultatively anaerobic bacteria. In one of the studies conducted on SSI in Surgical Clinic of Sismanoglion General Hospital of Athens, 9.8% patients were found to suffered from *K. pneumonia* infections [32].

VBNC bacteria refers to the bacteria that remain in a state of very low metabolic activity due to environmental stress i.e. different from starvation, do not divide for long time but are alive and does not appear in conventional culture [14]. These VBNC bacteria, although did not appear in the culture media but, can retain their cellular functions for long time and again can reproduce later and create a health risk [12]. As this is a tertiary care hospital, most of the patients come here after long term antibiotic treatment from primary health care hospitals, hence most of the pathogenic bacteria might be transformed into viable but non-culturable (VBNC) state due to either stress, biofilm or spore formation. In one of the review articles, several culturable bacterial species to transform to VBNC state in stress conditions are reported [33]. Many research articles have reported the presence of these VBNC bacteria in patient samples, which can be identified by PCR assay [12]. As per literature, methods based on culture will not detect these VBNC bacteria, even if these bacteria can be cultured and will grow in specific conditions such as; within an optimum range of temperature, osmotic conditions, pH, and in the presence of the correct nutrients. Some studies also reported that, pathogenic bacteria such as *Bacillus sp., Pseudomonas sp., Staphylococcus sp.* turn to slow or non-growing bacterial cells called “persisters” to survive from lethal dose of antibiotics, hence they did not come in culture plate within 48 h of incubation corroborating with previous studies [34].

Most of the SSIs due to anaerobic bacteria are derived from the host’s own endogenous flora, with few exceptions like *Clostridium spp*. These endogenous obligatory anaerobic bacteria play a vital role in preventing the colonization of several pathogenic and exogenous microbial populations, but due to some structural or functional defects in the mucous layer or obstructions become pathogenic [35]. The predominant anaerobic bacteria which are involved in SSI include *Bacteroides* fragilis group, *Prevotella* spp., *Porphyromonas* spp., *Fusobacterium* spp., *Peptostreptococcus* spp., *Clostridium* spp. and *Actinomyces*spp. [35]. In this study, we have reported the presence of some anaerobic bacteria such as; *Bacteroides, Fussobactrium spp., Sneathia spp., Peptoniphilus spp. and Peptostreptococcus spp*. in the wound swab/ aspirates of culture negative SSI patients attending the gastrointestinal and orthopaedic department. As there is no anaerobic culture set up available in this medical college, even though anaerobic bacteria were present in some samples, they cannot grow on culture plate in aerobic conditions [36]. This emphasises set up both aerobic and anaerobic microbial culture facility to support the growth of both aerobic and anaerobic bacteria in clinical samples to provide better patient care. Detection of unculturable bacteria in 13.4% (13 out of 97) of culture negative SSI samples draws attention for developing suitable laboratory media/ conditions to support the growth of these currently uncultured bacteria. This is a very challenging area of research that needs clear understanding of the metabolic pathway of these bacteria.

## Conclusions

Despite development in surgical and sterilization techniques and use of prophylactic antimicrobials, SSIs continue to pose clinical challenge. SSI samples of patients with no growth in culture after 48 h of incubation further complicates the situation. Certain experimental measures can be taken to improve the diagnosis of such culture negative samples. First culture plates should be allowed to incubate for an additional 3–4 days, which will allow the growth of fastidious bacteria if present. Second as anaerobic culture system is rarely available in the microbiology laboratory in Indian set up, it should be made available so that anaerobic bacteria can be identified in culture. Third as several unculturable bacteria and VBNC bacteria are responsible of culture negative SSI, molecular diagnosis by 16Sbroad range PCR assay can be employed for identifying such organisms in sample to help the clinicians in prescribing appropriate antibiotic to the patient. The study can further be extended to detect the antibiotic sensitivity /resistance pattern and study epidemiology of VBNC, anaerobes and unculturable bacteria using 16S broad range PCR assay.

## Data Availability

All the data related to the manuscript is available in NCBI. All the nucleotide sequences were submitted to the NCBI data bank and obtained the Accession numbers. So, these are available from NCBI and given in Table 1.

## Abbreviations

SSI: Surgical site Infections
PCR: Polymerase chain reaction

## References

[1] Reichman DE, Greenberg JA (2009). Reducing surgical site infections: a review. Rev Obstet Gynecol.

[2] Smyth ET, Emmerson AM (2000). Surgical site infection surveillance. J Hosp Infect.

[3] Mawalla B, Mshana SE, Chalya PL, Imirzalioglu C, Mahalu W (2011). Predictors of surgical site infections among patients undergoing major surgery at Bugando Medical Centre in Northwestern Tanzania. BMC Surg.

[4] Allegranzi B, Nejad SB, Combescure C, Graafmans W, Attar H, Donaldson L (2011). Burden of endemic health-care-associated infection in developing countries: systematic review and meta-analysis. Lancet.

[5] Arora A, Bharadwaj P, Chaturvedi H, Chowbey P, Gupta S, Leaper D (2018). A review of prevention of surgical site infections in Indian hospitals based on global guidelines for the prevention of surgical site infection, 2016. J Patient Saf Infect Control.

[6] European Centre for Disease Prevention and Control. Surveillance of surgical site infections in Europe 2010e2011.Stockholm: ECDC. 2013.

[7] Spagnolo AM, Ottria G, Amicizia D, Perdelli F, Cristina ML (2013). Operating theatre quality and prevention of surgical site infections. J Prev Med Hyg.

[8] Arumugam M, Raes J, Pelletier E, Le PD, Yamada T, Mende DR (2011). Enterotypes of the human gut microbiome. Nature.

[9] Reddy BR (2012). Management of culture-negative surgical site infections. J Med Allied Sci.

[10] Lee JC, Baek MJ, Choi SW, Kwon SH, Kim KH, Park SY (2018). Retrospective analysis of culture-negative versus culture-positive postoperative spinal infections. Medicine (Baltimore).

[11] Butler-Wu SM, Burns EM, Pottinger PS, Magaret AS, Rakeman JL, Matsen FA, Cookson BT (2011). Optimization of periprosthetic culture for diagnosis of Propionibacterium acnes prosthetic joint infection. J Clin Microbiol.

[12] Barer M, Bogosian G, Steck T (2004). The viable but nonculturable concept, bacteria in urine samples, and Occam's razor. J Clin Microbiol.

[13] Mukamolova GV, Kaprelyants AS, Kell DB, Young M (2003). Adoption of the transiently non-culturable state—a bacterial survival strategy?. Adv Microb Physiol..

[14] Zhao X, Zhong J, Wei C, Lin CW, Ding T (2017). Current perspectives on viable but non-culturable state in foodborne pathogens. Front Microbiol.

[15] Edmiston C, Krepel C, Seabrook G, Jochimsen W (2002). Anaerobic infections in the surgical patient: microbial etiology and therapy clinical infectious diseases. Clin Infect Dis.

[16] Rasnake M, Dooley D (2007). Culture-negative surgical site infections. Surg Infect.

[17] Stewart E (2012). Growing unculturable bacteria. J Bacteriol.

[18] Cooper R (2013). Surgical site infections: epidemiology and microbiological aspects in trauma and orthopaedic surger. Int Wound J.

[19] Lilani SP, Jangale N, Chowdhary A, Daver GB (2005). Surgical site infection in cleanand clean contaminated cases. Indian J Med Microbiol.

[20] Arya M, Arya PK, Biswas D, Prasad R (2005). Antimicrobial susceptibility pattern of bacterial isolates from post-operative wound infections. Indian J Pathol Microbiol.

[21] Golia S, Kamath ASB, Nirmala AR (2014). A study of superficial surgical site infections in a tertiary care hospital at Bangalore. Int J Res Med Sci.

[22] Bhave P, Kartikeyan S, Ramteerthakar M, Patil N (2016). Bacteriological study of surgical site infections in a tertiary care hospital at Miraj, Maharashtra state. India Int J Res Med Sci.

[23] Dellinger E (2011). Surgical site infections.

[24] Mundhada AS, Tenpe S (2015). A study of organisms causing surgical site infections and their antimicrobial susceptibility in a tertiary care government Hospital. Indian J Pathol Microbiol.

[25] Giacometti A, Cirioni O (2000). Epidemiology and microbiology of surgical wound infections. J Clin Microbiol.

[26] Pochhammer J, Weller M-P, Schäffer M (2016). Polihexanide for prevention of wound infection in surgery. Is the contact time essential? POLIS-trial: a historic controlled, clinical pilot trial. Wound Med..

[27] Loss G, Simões PM, Valour F, Cortês MF, Gonzaga L, Bergot M (2019). Staphylococcus aureus small colony variants (SCVs): news from a chronic prosthetic joint infection. Front Cell Infect Microbiol.

[28] Edmiston CE, Walker AP, Krepel CJ, Gohr C (1990). The nonpuerperal breast infection: aerobic and anaerobic microbial recovery from acute and chronic disease. J Infect Dis.

[29] Thilesen C, Nicolaidis M, Lökebö J, Falsen E, Jorde A, Müller F (2007). Leptotrichiaamnionii, an emerging pathogen of the female urogenital tract. J Clin Microbiol.

[30] Sintchenko V, Jelfs P, Sharma A, Hicks L, Gilbert G, Waller C (2000). Massilia timonae: an unusual bacterium causing wound infection following surgery. Clin Microbiol Newsl.

[31] Brook I (1998). Aerobic and anaerobic microbiology of infections after trauma in children. J Accid Emerg Med.

[32] Alexiou K, Drikos I, Terzopoulou M, Sikalias N, Ioannidis A, Economou NA (2017). Prospective randomised trial of isolated pathogens of surgical site infections (SSI). Ann Med Surg.

[33] Li L, Mendis N, Trigui H, Oliver J, Faucher S (2014). The importance of the viable but non-culturable state in human bacterial pathogens. Front Microbiol.

[34] Wang W, Chen J, Chen G, Du X, Cui P (2015). Transposon mutagenesis identifies novel genes associated with *Staphylococcus aureus* persister formation. Front Microbiol.

[35] Ananth-Shenoy P, Vishwanath S, Targain R, Shetty S (2016). Sunil-Rodrigues G et al Anaerobic infections in surgical wards—a two year study. Iran J Microbiol.

[36] Edmiston C, Krepel C, Seabrook G, Jochimsen W (2002). Anaerobic infections in the surgical patient: microbial etiology and therapy. Clin Infect Dis.

